# *In Silico*, *In Vitro* and *Ex Vivo* Evaluation of the Antihyperglycaemic, Antioxidant and Cytotoxic Properties of *Coccinia grandis* L. Leaf Extract

**DOI:** 10.17113/ftb.62.02.24.8162

**Published:** 2024-06

**Authors:** Pawan Prabhakar, Sayan Mukherjee, Ankit Kumar, Rahul Kumar Rout, Suraj Kumar, Deepak Kumar Verma, Santanu Dhara, Pavuluri Srinivasa Rao, Mrinal Kumar Maiti, Mamoni Banerjee

**Affiliations:** 1 Bio-Research Laboratory, Rajendra Mishra School of Engineering Entrepreneurship, Indian Institute of Technology Kharagpur, Kharagpur 721 302, West Bengal, India; 2 School of Medical Science and Technology, Indian Institute of Technology Kharagpur, Kharagpur 721 302, West Bengal, India; 3 Agricultural and Food Engineering Department, Indian Institute of Technology Kharagpur, Kharagpur 721 302, West Bengal, India; 4 Department of Biotechnology, Indian Institute of Technology Kharagpur, Kharagpur 721 302, West Bengal, India

**Keywords:** diabetes mellitus, *Coccinia grandis*, phytochemicals, extraction optimisation, molecular docking, enzyme inhibition, cytotoxicity

## Abstract

**Research background:**

*Coccinia grandis* L. is traditionally used for the treatment of diabetes mellitus. Since the scientific evidence and mechanism of action have not yet been extensively investigated, this study aims to evaluate the antidiabetic and cytotoxic effects together with the optimisation and development of a scale-up process design for higher yields of bioactive phytocompounds from *C. grandis.*

**Experimental approach:**

The *in silico* study was conducted to predict the binding affinity of phytocompounds of *C. grandis* for α-amylase and α-glucosidase enzymes involved in the pathophysiology of diabetes with pharmacokinetic assessment. Response surface methodology was used to determine the optimum total phenolic content (TPC), total flavonoid content (TFC), total tannin content (TTC) and antioxidant activities (DPPH and FRAP) in 17 different experimental runs in which the parameters of microwave-assisted extraction such as temperature (50–70 °C), power (400–1000 W) and time (15-45 min) were varied. The phytocompounds were purified and identified using column chromatography, thin-layer chromatography (TLC), UV-visible spectroscopy, Fourier transform infrared spectroscopy (FTIR) and liquid chromatography-mass spectrometry (LC-MS). The *in vitro* antidiabetic activity was determined by α-amylase and α-glucosidase enzymatic inhibitory assays, while cytotoxic investigations were done by measuring haemolytic activity, 3-[4,5-dimethylthiazol-2-yl]-2,5-diphenyl tetrazolium bromide (MTT) and chorioallantoic membrane (CAM) assays.

**Results and conclusions:**

The reported major bioactive compounds have shown an excellent binding affinity for α-amylase and α-glucosidase enzymes in the range of −14.28 to −36.12 kJ/mol with good pharmacokinetic properties and toxicities ranging from low to medium. The bioactive constituents such as total phenols, total flavonoids, total tannins and antioxidant activities such as DPPH and FRAP were found to be high and dependent on the optimised microwave-assisted extraction parameters such as temperature, time and power: 55 °C, 45 min and 763 W, respectively. Sixteen compounds were identified by FTIR and LC-MS spectra in the plant sample after preliminary identification, purification and TLC. The percentage of enzyme inhibition depended on the concentration of the extract (7.8–125.0 µg/mL) and was higher than that of acarbose. The haemolytic activity was in accordance with ISO standards and low toxicity was observed in the MTT and CAM assays in the range of 7.8−125.0 µg/mL, suggesting its potential use as an antidiabetic drug and for functional food development.

**Novelty and scientific contribution:**

The results of the study open up new opportunities for researchers, scientists and entrepreneurs in the food and pharmaceutical sectors to develop antidiabetic foods and medicines that help diabetics to better control their condition and maintain overall health.

## INTRODUCTION

Diabetes mellitus, a common medical condition, comprises a series of permanent metabolic disorders characterised by the presence of hyperglycaemia or increased glucose levels in the blood. The above symptoms are the result of compromised insulin secretion, insulin action or a combination of both (*1*). Prolonged elevated blood glucose levels can lead to severe detrimental effects on various physiological systems, such as renal dysfunction, myocardial infarction, cerebrovascular accidents, retinal damage and the formation of opacities in the lens of the eye (*2*). The global prevalence of diabetes is on an upward trend, which means that an increasing number of individuals worldwide are affected by this metabolic disorder. Based on the International Diabetes Federation (IDF) projections, it is estimated that the global population with diagnosed and undiagnosed diabetes will reach approx. 536.6 million people in 2021. Furthermore, this number is expected to increase by 46 % over the next 24 years, reaching a staggering 783.2 million by 2045 (*3*). Enzyme inhibitors such as acarbose, miglitol and voglibose are used to treat postprandial hyperglycaemia (PPHG). However, both miglitol and voglibose only inhibit α-glucosidase, while acarbose effectively inhibits both α-amylase and α-glucosidase (*4*). Because of the adverse effects on the gastrointestinal tract that these inhibitors can cause, long-term medication is not recommended. Drugs of natural origin have enormous benefits in treating chronic disorders with fewer or no side effects. They are compatible with human physiology and are widely accepted by the population (*5*, *6*). Therefore, researchers are focusing on both α-amylase and α-glucosidase inhibitors of natural origin, such as plants, microbes and marine animals.

The leaves and fruits of *Coccinia grandis* L. are used by various tribal populations as ethnomedicinal agents and food ingredients for the treatment of diabetes. Despite the widely recognised physiological effects of *C. grandis* and its involvement in various health and disease processes, there has been a notable lack of research efforts addressing the formulation of therapeutic interventions using the phytoconstituents of this plant species. Previous studies have shown the potential advantages of *C. grandis* leaf extract for the treatment of diabetes (*7*). Nevertheless, there is a need for more scientific literature that can provide a comprehensive understanding of the exact mechanism underlying the antidiabetic effects and assessment of toxicity.

Given the above, the aim of this research is to investigate *in silico*, *in vitro* and *ex vivo* the potential of the phytocomponents of *C. grandis* in the treatment of diabetes. This was achieved by investigating their ability to inhibit the enzymes α-amylase and α-glucosidase and by studying the absorption, distribution, metabolism and excretion (pharmacokinetics) of the drug and its potential to cause harm to cells (cytotoxicity). The overall goal is to expand academic, scientific and industrial efforts related to the development of antidiabetic foods and drugs as well as their formulations.

## MATERIALS AND METHODS

### In silico analysis

The software used in this study was AutoDock Tools, v. 1.5.7 (*8*), Discovery Visual 2020 software (*9*) and Open Babel, specifically the Open Babel GUI (graphical user interface), v. 2.4.1 (*10*). The online web server used in this study was ADMETlab, v. 2.0 (*11*).

### Chemicals and reagents

Ethanol (99.9 % purity), methanol, sodium carbonate, aluminium chloride and iron(III) chloride (all 99 % purity), 37 % hydrochloric acid and sodium phosphate (96 % purity) were obtained from Merck, Mumbai, Maharashtra, India. Folin-Ciocalteu reagent and 3-[4,5-dimethylthiazol-2-yl]-2,5-diphenyl tetrazolium bromide (MTT) assay kit (both 98 % purity) were purchased from HiMedia, Thane, Maharashtra, India. Highly purified standards, namely quercetin, stigmasterol and kaempferol (all 95 % purity), were purchased from Sigma Lifesciences, Bangluru, Karnataka, India. Silica gel with a mesh size of 60-120 and a thin-layer chromatography (TLC) plate with an F254 coating were obtained from Merck, Darmstadt, Germany. Trolox (97 % purity) and soy lecithin (95 % purity) were obtained from Sigma-Aldrich, Merck, Bangaluru, Karnataka, India. Gallic acid (99 %), tannic acid (98 %), Trolox (97 %), 2,4,6-tripyridyl-*S*-triazine (TPTZ, 99 %), porcine pancreatic α-amylase, α-glucosidase, dimethyl sulfoxide (DMSO, 99 %), acarbose (95 %), starch solution, 3,5-dinitrosalicylic acid (DNSA, 99 %) and *p*-nitrophenyl-β-d-galactopyranoside (*p-*NPG, 99 %) were purchased from SRL Chemicals, Mumbai, Maharashtra, India. Culture media such as Dulbecco's modified Eagle medium (DMEM) were obtained from Gibco, Life Technologies Ltd., Paisly, UK and reagents of analytical grade were purchased from Merck, Mumbai, India.

### Molecular docking studies

Based on previous scientific research and comprehensive databases, 19 prominent bioactive phytocompounds with both antidiabetic and antioxidant properties have been successfully identified. Acarbose, a commonly used therapeutic agent, was acquired from PubChem (*12*) in sdf format and subsequently converted to pdb format using Open Babel software (*10*). The application of Lipinski's rule of five in this context was facilitated by the advanced computational resources accessible at the bioinformatics and computational biology supercomputing facilities at IIT Delhi, India. The crystal structures of α-amylase and α-glucosidase enzymes were deposited in the protein database by Tan *et al.* (*13*) and Lodge *et al.* (*14*) at a resolution of 1.35 and 1.90 Å, respectively, and used for the analysis. Discovery Studio (*9*) was used for the removal of water molecules, heteroatoms and ligands. AutoDock (*8*) was used to introduce gastgeiger charges to the central grid. The central grid was configured to rejuvenate the active site pockets. The values for the central grids and the total number of points are shown in Table S1. To achieve maximum accuracy in the docking procedures, we maintained the exhaustiveness level at 100. First, a molecular docking procedure was used to investigate the interaction between acarbose and enzymes. Then, the phytochemical compounds were introduced into the system. AutoDock Vina (*8*) performed the docking process with its command mode, while the Discovery Studio (*9*) facilitated the visualisation of the molecular structure.

### Pharmacokinetic analysis

The pharmacokinetic analysis showed different values for absorption, distribution, metabolism, excretion and toxicity. The water pollution and ecological imbalance were assessed using the LC_50_ (48-hour exposure of *Tetrahymena pyriformis*, 96 h exposure of fathead minnow and 48 h exposure of *Daphnia magna*) as a means of testing the chemical concentration present in water. The expected concentration of phytocompounds was predicted to be relatively low, suggesting that they may pose a lower risk to aquatic ecosystems (*15*, *16*). The bioconcentration factor was used as a means of assessing potential hazards to human health associated with the food chain.

#### Optimisation of MAE parameters

The leaf samples were taken and collected from plants of the agricultural land of the Agriculture and Food Engineering Department, Indian Institute of Technology Kharagpur, Kharagpur, India, at coordinates 22.31°N and 87.31°E. After authentication, the leaves were washed, air-dried for 45 days, sealed and preserved. Based on previous research, 80 % ethanol and the use of microwave-assisted extraction (MAE) were found to be the most effective solvent and extraction method for the extraction of phytocompounds such as polyphenols. The extraction efficiency during the MAE process can be influenced by temperature, time, power and other factors to maximise the desired results.

#### Experimental design

As in the study conducted by Dahmoune *et al.* (*17*), a three-level Box Behnken design (BBD) with three factors was used to optimise the extraction variables. The independent variables in the study were temperature, power and time, which ranged from 50 to 70 °C, 400 to 100 W and 15 to 45 min, respectively. Preliminary experiments and previous research determined the limits of the variables involved. The solvent-to-sample ratio was determined to be 8:1 and ethanol (80 %) was used as the solvent. Total phenolic content (TPC), total flavonoid content (TFC), total tannin content (TTC), 2,2-diphenyl-1-picrylhydrazyl (DPPH) and Fe(III) reducing antioxidant power (FRAP) were measured as the antioxidant capacities of the plant extracts. The Design Expert software, v. 11.12.0 (*18*) and the BBD with five central points were used in this study. The response variables were modelled using a second-order polynomial equation:


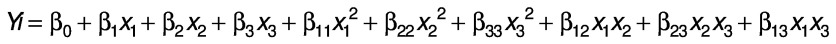
 /1/

where Yi represents the response variable, β_0_ denotes the constant term, β_1_, β_2_ and β_3_ are the regression coefficients for the linear terms, β_11_, β_22_ and β_33_ are the regression coefficients for the quadratic terms, while β_12_, β_23_ and β_13_ represent the regression coefficients for the interaction terms. The independent variables x_1_, x_2_ and x_3_ correspond to temperature, power and time, respectively. Three experimental runs were carried out and the mean values were analysed. The appropriateness of the model was verified based on R^2^ value, p-value, CV-value and the lack of fit.

### Determination of phytocompounds

#### Total phenolic content

The TPC in the leaf extract of *C. grandis* was determined using the method proposed by Do *et al.* (*19*), with some adjustments. The plant extracts and gallic acid solutions were prepared at different concentrations (100–500 μg/mL). These solutions were then used to construct a calibration curve based on a linear equation. A volume of 0.5 mL of crude extracts at a concentration of 0.1 mg/mL was added to a test tube. Subsequently, 0.5 mL of distilled water and 1 mL of the Folin-Ciocalteu reagent were mixed. After 3 min, a volume of 2 mL of 20 % sodium carbonate solution was added to the mixture. The mixture was placed in the dark at 25 °C for 60 min. The absorbance of the sample was then measured at *λ*=765 nm using a UV-Vis spectrophotometer (UV 1601; Shimadzu, Kyoto, Japan). The TPC was expressed in mg of gallic acid equivalents (GAE) per g of dry mass. The experiment was carried out in triplicate (*N*=3), and the mean values were calculated together with the corresponding standard deviations.

#### Total flavonoid content

The TFC w*a*s determined using the aluminium chloride colorimetric method following the protocol proposed by Sen *et al.* (*20*) with minor modifications. First, 3 mL of distilled water was mixed with 1 mL of each respective extract sample, then 0.2 mL of 10 % aluminium chloride solution was added. After a 10-minute incubation, 0.2 mL of 1 M potassium acetate and 6 mL of distilled water were added. The solution was incubated for 30 min and then the absorbance at *λ*=415 nm was measuredusing a UV-Vis spectrophotometer (UV 1601; Shimadzu). Quercetin solutions (50–500 µg/mL) were prepared and used to determine a standard calibration curve. The results were expressed in mg of quercetin equivalents (QE) per g of dry mass. The experiment was performed in triplicate (*N*=3), and the mean values were calculated together with the corresponding standard deviations.

#### Total tannin content

The TTC assay was performed using the method proposed by Kumar *et al.* (*21*), with some adjustments. First, a volume of 1 mL of the diluted extracts was added to a solution containing 0.5 mL of the Folin-Ciocalteu reagent. This mixture was then allowed to stand without mixing for 3 min. A volume of 1 mL of 7.5 % sodium carbonate was added to the mixture and incubated for 30 min at 25 °C in the dark. Tannic acid was used as a reference standard and the standard calibration curve was determined in a concentration range of 100–500 μg/mL. The absorbance was measured at *λ*=593 nm using a UV-Vis spectrophotometer (UV 1601; Shimadzu). The tannin was determined as tannic acid equivalents (TAE) in mg per g of dry mass. The experiment was done in triplicate (*N*=3), and the mean values were calculated together with the corresponding standard deviations.

### Determination of antioxidant activities

#### DPPH antioxidant activity

The method developed by Marinova and Batchvarov (*22*) was used with some modifications to determine the DPPH antioxidant activity of the solvent extract of *C. grandis*. A volume of 1 mL of the plant extract was diluted with 2 mL of methanol in a test tube and then 1 mL of 0.004 mM DPPH-methanol solution was added. The mixture was then incubated in the dark for 30 min. The absorbance was measured at *λ*=517 nm using a UV-Vis spectrophotometer (UV 1601; Shimadzu). The radical scavenging capacity of the sample was determined using the following formula:


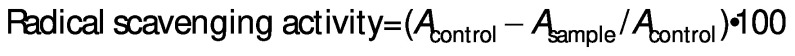
 /2/

The experiment was done in triplicate (*N*=3), and the mean values were calculated together with the corresponding standard deviations.

#### FRAP antioxidant activity

The experiment was conducted to measure the reducing antioxidant power according to Benzie and Strain (*23*) with minor modifications. The FRAP reagent was prepared with 150 mL of acetate buffer, 5 mM TPTZ dissolved in 20 mM HCl and 10 mM iron(III) chloride hexahydrate in a volume ratio of 10:1:10. After adding 1 mL of the freshly prepared reagent to 2 mL of each of the diluted plant extracts, the mixture was thoroughly homogenised. The mixture was then incubated at 37 *°*C for 30 min. The absorbance of resulting vibrant blue colour of solution was measured at *λ*=593 nm using a UV-Vis spectrophotometer (UV 1601; Shimadzu). A reagent blank consisting of 2 mL of FRAP reagent and 1 mL of deionized water was used as a reference. A calibration curve was determined with Trolox at concentrations from 1 to 5 mg/mL and FRAP was expressed in mg of Trolox equivalents (TE) per g of dry mass of plant sample. The experiment was done in triplicate (*N*=3), and the mean values were calculated together with the corresponding standard deviations.

### Preliminary phytochemical screening, purification and thin-layer chromatography of plant extracts

The phytochemical screening of bioactive compounds in plant extracts using appropriate reagents is one of the first detection methods for primary and secondary metabolites. Previous studies have confirmed the use of established methods to assess the phytochemical composition of plant leaf extracts (*24*-*26*). These methods were used for initial screening of phytocompounds. Column chromatography was used with some modifications to analyse the unprocessed leaf extracts as reported by Chandrappa *et al.* (*27*). A slurry was prepared by combining silica gel with a mesh size ranging from 230 to 400 with *n*-hexane. Again, the silica gel was densely packed with the slurry to prevent any sinking. The column was primed with a blank run and then flushed with *n*-hexane to optimise the solvent flow rate. A conical flask was placed at the base of the column in close proximity to the end point to collect the eluate. The material that was obtained was then deposited in the column and the mobile phase was selected by TLC with the specified combination of solvents. In the experiment, toluene, ethyl acetate, methanol and formic acid were used as solvents in a volume ratio of 75:25:25:6. The migration of the desired compound was monitored using a silica gel plate. A total of seven fractions were obtained after using an isocratic mobile phase consisting of toluene, ethyl acetate, methanol and formic acid in a volume ratio of 200:64:64:16. Each segment had a volumetric capacity of 56 mL. The aliquots were collected, concentrated using a rotary evaporator (Stuart WW-16340-59; Cole-Parmer India, Mumbai, India) and then subjected to TLC analysis. A mobile phase consisting of toluene, ethyl acetate, methanol and formic acid in a volume ratio of 5:1.6:1.6:0.4 was used for chromatographic separation. The resulting spots were then compared with the reference standards of quercetin and kaempferol. Fractions 2 and 3 were mixed, and the resulting mixture was subjected to column chromatography again for comparison with the quercetin and kaempferol standards.

### Identification and characterisation of bioactive phytocompounds

#### UV-visible spectroscopy

This study was conducted to analyse the UV-Vis spectrum of the plant extracts. A wavelength range of 200 to 700 nm was used for the study according to the methodology proposed by Sharma *et al.* (*28*). The reference compounds, pure standard quercetin and kaempferol, were used for their respective spectra. The spectra were obtained using a quartz cuvette with a path length of 10 mm and a UV-Vis spectrophotometer (UV 1601; Shimadzu).

#### Fourier transform infrared spectroscopy

The identification of the functional groups and their corresponding vibrational frequencies in the leaf extract was performed using Fourier transform infrared spectroscopy (FTIR) (Nicolet 6700 spectrophotometer; Thermo Fisher Scientific, Waltham, MA, USA). The active functional groups of the components were categorised according to their respective peak frequencies (*29*), as they are distinctive as ’fingerprint’ of an organic molecule (*30*). The sample was mixed with potassium bromide and analysed by FTIR. The observed absorbance band in the FTIR spectra of *C. grandis* corresponds to wavenumbers from 3500 to 400 cm^-1^.

#### Liquid chromatography-mass spectrometry of plant extracts

The liquid chromatography-mass spectrometry (LC-MS) analysis of the *C. grandis* extract was performed according to Al-Madhagy *et al.* (*31*) with minor adjustments. After dilution of the extract by a 100-fold factor, the samples were analysed using an LC-MS system (Waters, Milford, MA, USA). The LC-MS system was equipped with a photodiode array detector operating at a wavelength of 250 nm. The analytical C18 column (Waters) was used as the stationary phase. The device consists of a combined integrated and automated fraction collector. The Quattro micro^TM^ Application Programming Interface (API) in conjunction with the updated mass-Lynx 4.7 software was used to control the instrument and collect the data. A mobile phase consisting of an aqueous solution containing 0.1 % formic acid (A) and methanol acidified with 0.1 % formic acid (B) was used for gradient elution at a flow rate of 0.5 mL/min. Gradient elution began with a mixture of 90 % solvent A and 10 % solvent B, which was maintained from 0 to 45 min. The composition was then changed to 100 % solvent B from 45 to 55 min. The eluent was then reverted to a mixture of 90 % solvent A and 10 % solvent B from 55 to 55.5 min, and this composition was maintained until 60 min. Mass spectra were recorded in the mass-to-charge ratio (*m/z*) range of 50–2000 at 400 °C and a nebulising gas flow rate of 10 L/min. Both negative and positive ionization modes were used and a chromatogram was recorded at a wavelength of 350 nm.

### Determination of in vitro enzyme inhibitory potential

#### Inhibition of α-amylase

The enzyme inhibition methods employed by Nair *et al.* (*32*) were used with slight modifications to assess the α-amylase inhibitory activity of a plant extract. Two test tubes were prepared using a 5 % DMSO solution. In one test tube, 1 mL of the plant extract was combined with 1 mL of acarbose. The other test tube contained 1 mL of 20 mM sodium phosphate buffer (pH=6.8) with 40 µL of porcine pancreatic amylase (0.5 mg/mL). The plant extract and the acarbose mixture were then added to the second test tube at various concentrations ranging from 7.8 to 125.0 µg/mL. After 15 min at 25 °C, a 0.5 % starch solution was added to the solution. The resulting mixture was then incubated to a further 20 min. Then, 1 mL of DNSA solution was added to terminate the ongoing chemical reaction. The test tubes were then immersed in a temperature-controlled water bath for 5 min, followed by a cooling period. An aliquot of 5 mL of distilled water was then added to each test tube to achieve dilution. The absorbance of the samples was measured at *λ*=540 nm using a UV-Vis spectrophotometer (UV 1601; Shimadzu). The control solution consisted of starch, enzyme, DMSO and DNSA, while starch was used to prepare the blank sample. Acarbose was used as a reference compound and the percentage of inhibition of the α-amylase enzyme by the plant extract was determined using Eq. 2. The experiment was performed in triplicate (*N*=3) and the mean values were calculated together with the corresponding standard deviations.

#### Inhibition of α-glucosidase

In separate test tubes, a volume of 1 mL of plant extract and acarbose was diluted in a 5 % DMSO solution at different concentrations from 7.81 to 125 µg/mL. The method used to evaluate the inhibitory activity of the α-glucosidase enzyme was based on the approach described by Alqahtani *et al.* (*33*) with slight modifications. A solution containing 1 mL of α-glucosidase (1 mg/100 mL) in 100 mM phosphate buffer (pH=6.8) was added to both the extracts and acarbose. The resulting mixture was incubated at 37 °C for 15 min. The chemical process was then stopped by adding 2.5 mL of a 0.1 M sodium carbonate solution to a mixture containing 500 µL of a 5 mM *p*-NPG prepared in a 100 mM phosphate buffer (pH=6.8). After 30 min at 37 °C, the absorbance at *λ*=415 nm was measured with UV-Vis spectrophotometer (UV 1601; Shimadzu). The inhibition percentage was calculated using Eq. 2, with acarbose as the reference standard. The experiment was performed in triplicate (*N*=3), and the mean values were calculated together with the corresponding standard deviations.

### Determination of in vitro and ex vivo toxicity

#### Haemocompatibility assay

The *in vitro* haemocompatibility tests evaluate the effects of medical devices or substances that come into contact with blood or its components. The methodology proposed by Sulaiman and Gopalakrishnan (*34*) was used, with minor modifications. Vacutainers containing a mixture of sodium citrate and heparin sodium in a volume ratio of 1:9 were used to collect 5 mL of fresh human blood from a healthy individual. After centrifuging the sample for 15 min at 1377×*g* using the centrifuge (model 5418; Eppendorf, Hamburg, Germany), the residue was found to consist predominantly of red blood cells. This method was used to effectively separate the plasma from the sample. Phosphate-buffered saline (PBS) was used to rinse and dilute the concentrated red blood cell suspension. A volume of 200 μL of the sample extract was added to 800 μL of red blood cell suspension in PBS to perform the tests. Positive and negative controls were prepared by combining 0.2 mL of diluted blood with 0.8 mL of deionized water and PBS, respectively. After a 3-hour incubation at 37 °C, all tubes were centrifuged at 2152×*g* for 10 min (centrifuge model 5418; Eppendorf). The absorbance of the supernatant was measured at *λ*=545 nm using a UV-Vis spectrophotometer (UV 1601; Shimadzu). The percentage of haemolysis was determined using the following equation:


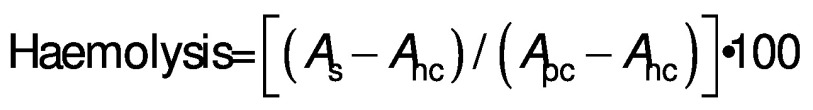
 /3/

where *A*_s_ is the absorbance of sample, *A*_pc_ and *A*_nc_ are the absorbance of the positive and the negative control, respectively. The experiment was performed in triplicate (*N*=3) and the mean values were calculated together with the corresponding standard deviations.

#### Cell viability assay

The MTT assay was used to evaluate the cytotoxicity of the samples and determine the cellular viability. The methods were adapted based on the research of Liu and Nair (*35*). The rabbit corneal L929 fibroblast cells were seeded at 4000 per well in 96-well plates for tissue culture at 37 °C. No cells were seeded in blank wells. After 24 h, *C. grandis* extract was added to the media at the concentrations of 7.8, 15.6, 31.2, 62.5 and 125.0 µg/mL) in triplicate. After 4 h at 37 °C, the cells were washed with PBS. After 48 and 72 h, the medium was discarded. Both the sample and control groups (cells without the sample) were then incubated in 0.5 mg/mL MTT solution. DMSO was added and stirred to dissolve the crystalline purple formazan. A microplate reader (Multiskan; Thermo Fisher Scientific) measured the absorbance at 595 nm and the percentage of cell viability was determined using the following equation:


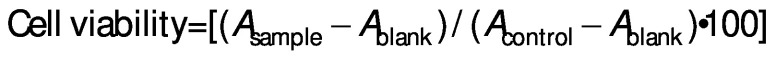
 /4/

The experiment was performed in triplicate (*N*=3) and the mean values were calculated together with the corresponding standard deviations.

#### Chorioallantoic membrane assay

The chorioallantoic membrane (CAM) assay is a simple and cost-effective *ex vivo* model for evaluating the toxicity of pharmacological samples. The toxicity assessment using the chick CAM model was performed using the methods described by Schneider-Stock and Ribatti (*36*), with some modifications. The fertilised chicken eggs came from a government poultry farm in Paschim Medinipur, India. Before incubation at 37 °C and a relative humidity of 80 %, all eggs were subjected to a mild sterilisation with ethanol (70 %). After two days of incubation, the eggs were divided into six different experimental cohorts, which contained a positive control. Five dilutions were prepared from the original stock solution of plant extract, resulting in concentrations from 7.8 µg/mL to 125.0 µg/mL. A precise opening of approx. 1 cm^2^ was made on the eggshell, on the side opposite the rounded edge. The initial chorionic membrane was carefully excised and washed with a PBS (pH=7.4). Experimental groups 1 to 5 were subjected to a treatment in which the extract was administered in different concentrations from 7.8 to 125.0 µg/mL. In contrast, the positive control group did not receive any form of treatment. The eggs were carefully sealed with paraffin tape and incubated at 37 °C and 80 % relative humidity. Photographs of the eggs were taken at specific time intervals of 48 and 72 h using a digital camera (Nikon D7500; Nikon Inc., Melville, NY, USA). The photographs were analysed using ImageJ software v. 1.54 (*37*).

### Statistical analysis

The experimental results were presented as mean value and standard deviation of the triplicate measurements. Statistical analyses of the experimental data were performed using the analysis of variance (ANOVA) and SPSS software v. 20.0 (*38*). A significant difference was found among all results at the p<0.05 and p<0.01 significance levels, as determined by Tukey’s HSD *post hoc* tests to measure significant differences in the data (*39*).

## RESULTS AND DISCUSSION

### In silico studies and pharmacokinetic assessment of Coccinia grandis phytocompounds

**Fig. S1:**
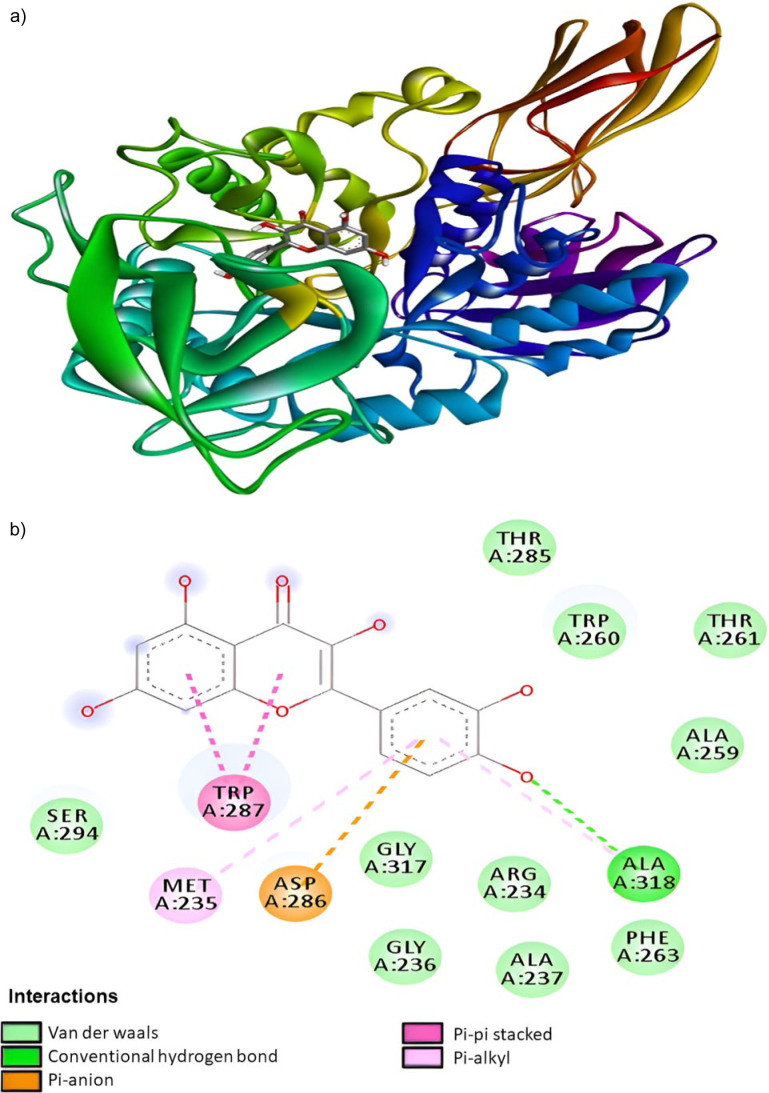
Molecular interactions between an enzyme-phytochemical complex in *Coccinia grandis* leaf extract: a) three-dimensional ribbon structure of α-amylase interacting with quercetin shows the binding position at the active site of the α-amylase, including the specific amino acid residues involved in the interaction, and b) two-dimensional structure illustrates the different types of bonds formed between the enzyme and quercetin, together with their respective bond orders, which range from 2.54 to 5.00 Å. A=side chain of α-amylase enzyme

The binding affinities of standard acarbose towards α-amylase and α-glucosidase were determined to be −21.86 and −34.02 kJ/mol, respectively (data not shown). To predict potential interactions between the α-amylase and the phytochemicals, a docking procedure was performed in which all 19 phytochemicals were positioned at the active site of the catalytic region of the enzyme. The results of the molecular docking analysis showed favourable affinities for the binding of the compounds to the α-amylase (Table 1). Quercetin showed a remarkable affinity as the average bond order ranged from 2.54 to 5.0 Å and the highest binding affinity of −32.76 kJ/mol was measured. Increased inhibitory activities were observed when stronger interactions, characterised by a decrease in bond order, occurred . This is primarily attributed to the presence of a larger number of Van der Waals interactions, together with the involvement of two hydrogen bonds (Asp286 and Ser294) and one pi-alkyl interaction (Trp287) (Fig. S1). The phytochemical compounds obtained from *Coccinia grandis* showed significant inhibitory properties and strong affinities for α-glucosidase, with binding energies ranging from −14.28 to −36.12 kJ/mol. Campesterol showed the most favourable binding affinity as evidenced by its binding energy of −36.12 kJ/mol. It also showed a notable binding affinity across a wide range of interactions and different range of bond orders. Limited *in silico* molecular docking studies of *C. grandis* have been performed to elucidate its potential antidiabetic activity against enzymes such as α-amylase and α-glucosidase, which are responsible for the increased glucose levels in the blood that lead to diabetes. Begum *et al.* (*40*) reported that the phytocompounds found in *C. grandis*, such as β-amyrin acetate and β-cryptoxanthin, exhibited PPAR-γ agonistic properties and had binding affinities of −17.51 and −23.1 kJ/mol, respectively. In a study conducted by Prabhakar *et al.* (*41*), molecular docking was performed on the dipeptidyl peptidase-4 (DPP4) enzyme. The researchers discovered that derivatives of phytosterols such as stigmasterol, which is found in *C. grandis*, have a binding affinity of −8.4 kcal/mol. Molecular docking thus proves to be a promising approach to identify potential compounds that could serve as primary therapeutics for the treatment of diabetes.

**Table 1:** List of identified phytochemicals from *Coccinia grandis* leaf extract with inhibitory activity against α-amylase and α-glucosidase, and their respective binding affinities

	Binding affinity/(kJ/mol)	
Phytocompound	α-amylase	α-glucosidase
Benzofuranone	−22.26	−21.42
Campesterol	−31.42	−36.12
Campocatechin	−31.42	−35.7
Coniferyl alcohol	−23.1	−21.42
Ethisterone	−30.24	−31.92
Ferulic acid	−24.36	−22.68
Furanone	−15.96	−14.28
Isosteviol	−32.76	−31.08
Kaempferol	−30.24	−29.82
Luteolin	−32.34	−31.92
Methyl caffeate	−32.34	−31.92
*p*-coumaric acid	−23.1	−23.1
Quercetin	−32.76	−29.4
Listroside	−32.34	−31.92
Lukianol	−32.34	−33.6
Oleuropein	−32.34	−31.92
Sinapic acid	−23.52	−23.52
Stigmasterol	−32.34	−35.28
Undecanol	−17.22	−15.54

The pharmacokinetic analysis of the 19 reported compounds was conducted with the exception of isosteviol, luteolin, listroside, oleuropein and quercetin. All phytocompounds showed a favourable potential for absorption by human intestinal tissue as indicated by the Caco-2 permeability prediction, which is commonly performed on human colon cancer cell lines (*42*). Most phytocompounds showed limited ability to cross the blood-brain barrier, which is a critical requirement for medications targeting peripheral systems to mitigate potential negative effects on the central nervous system (*15*). The compound P-glycoprotein plays a key role in drug efflux, *i.e.* it helps to remove drugs from cells. Consequently, activation of P-glycoprotein would lead to an increase in drug efflux, resulting in lower drug concentrations than required. This could potentially lead to treatment failure. Compounds such as ethisterone, camptothecin, isosteviol, kaempferol, quercetin, lukianol, sinapic acid and undecanol have a substrate affinity for P-glycoprotein. On the other hand, benzofuranone, coniferyl alcohol, ferulic acid, furanone, methyl caffeate and *p*-coumaric acid are less likely to be inhibitors of P-glycoprotein, as indicated in Table S2. As described by Xiong *et al.* (*43*), a variety of physiological methods have been used to evaluate the toxicity of medicinal phytocompounds. These techniques included the use of hERG blockers to assess cardiac safety, hepatotoxicity evaluation to examine potential liver damage, drug-induced liver injury (DILI) assessment, Ames toxicity test to determine mutagenic potential, acute oral toxicity studies in rats, skin sensitisation tests, carcinogenicity assays, eye irritation and corrosion testing and respiratory toxicity analysis. All phytocompounds had a low likelihood of acting as hERG blockers, while *p*-coumaric acid, camptothecin, bezofuranone, isosteviol and ferulic acid had a moderate to high likelihood of causing hepatotoxicity, and the bioactive phytocompounds had different degrees of hepatotoxicity, as shown in animal model tests. The Food and Drug Administration (FDA) has suggested that a moderate threshold for determining the harmful dose of chemicals to humans and low to moderate level of carcinogenicity should be established based on their ability to disrupt cellular metabolic processes or cause DNA damage (*44*). In the study, certain phytocompounds, namely campesterol, were found to have a rapid clearance rate from the body at 15 mL/(min·kg). In contrast, other phytocompounds such as quercetin, benzafuranone, luteolin and ferulic acid showed a comparatively slower clearance rate, ranging from 5 to 15 mL/(min·kg). The volume distribution was predicted to be in the medium range (between 0.3 and 0.9) and the majority of the phytocompounds had a moderate half-life and clearance rate (*45*). All compounds identified in the present study showed low to moderate toxicity to both biological organisms and the surrounding ecosystem, as indicated in the supplementary tables (Table S2, Table S3, Table S4, Table S5 and Table S6).

### Optimisation of microwave-assisted extraction and the effect of different conditions on the recovery of phytocompounds from C. grandis

**Fig. 1:**
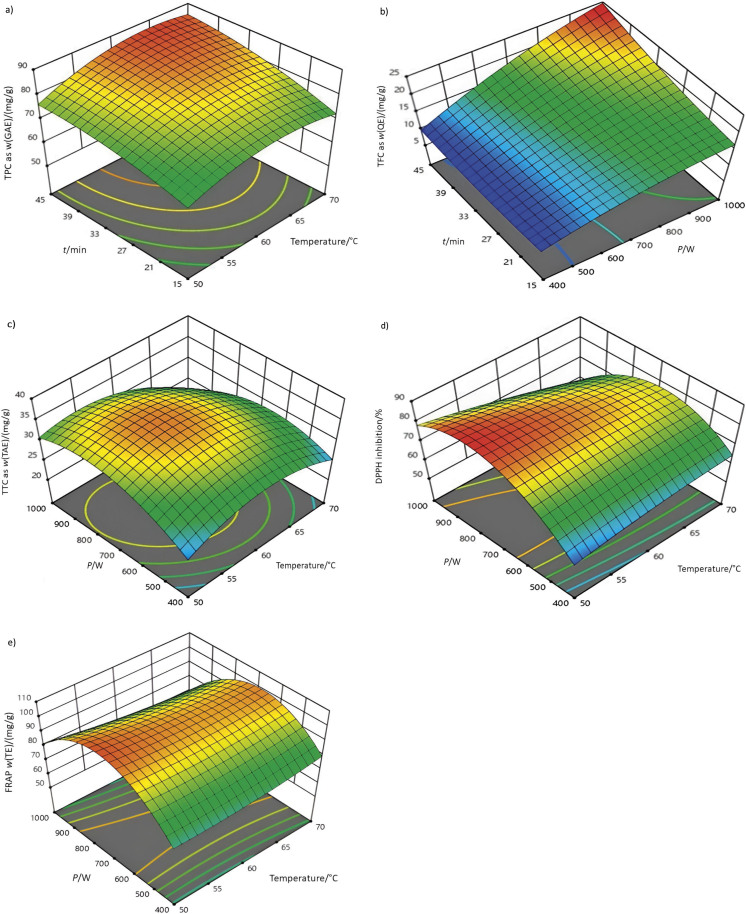
Response surface plots of the effect of microwave-assisted extraction conditions on different biochemical parameters in *Coccinia grandis* extract: a) total phenolic content (TPC), b) total flavonoid content (TFC), c) total tannin content (TTC), d) 2,2-diphenyl-1-picrylhydrazyl (DPPH) radical scavenging activity and e) Fe(III) reducing antioxidant power (FRAP)

The microwave-assisted extraction (MAE) was carried out under different conditions (temperature, power and time), and the response surface plots showing the effect of these conditions on various biochemical parameters are shown in Fig. 1. The response variables are shown in Table 2. The data for the fitting of the model for the response variables are shown in Table 3. The results of the statistical model fit show that the quadratic model represents the data most accurately. This finding was supported by a significant regression p-value (p<0.05), suggesting a strong relationship between the variables. Additionally, the lack of fit is deemed insignificant (p>0.05), indicating that the quadratic model adequately represents the underlying patterns in the data. The mass fraction of TPC, expressed as GAE, increased from 54.45 to 88.93 mg/g. The maximum TPC was obtained by MAE at 60 °C and a power of 700 W for 30 min. The minimum value was achieved at 60 °C and 1000 W for 15 min.

**Table 2:** Results of the study using the Box-Behnken design for the experimental setup and analysis of the response variables associated with the microwave-assisted extraction of *Coccinia grandis* leaves

Run	Temperature/°C	*P*/W	*t*/min	TPC as *w*(GAE)/(mg/g)	TFC as *w*(QE)/(mg/g)	TTC as *w*(TAE)/(mg/g)	DPPH inhibition/ %	FRAP as *w*(TE)/(mg/g)
1	60	700	30	88.93	14.13	33.36	83.96	100.10
2	60	700	30	81.28	13.84	33.97	78.59	95.15
3	60	700	30	81.73	13.91	36.51	83.85	101.99
4	60	400	45	76.98	10.09	29.69	63.75	80.13
5	50	700	45	73.73	22.93	32.83	86.39	106.87
6	70	400	30	68.24	9.88	25.31	66.63	79.95
7	60	700	30	80.73	18.8	37.57	82.10	100.55
8	70	1000	30	63.87	22.99	22.65	58.90	69.37
9	70	700	45	85.79	19.04	29.37	80.45	100.37
10	50	400	30	60.72	10.84	23.63	57.22	74.32
11	60	1000	45	68.95	24.16	30.24	72.85	73.95
12	60	1000	15	54.45	16.27	27.31	64.07	64.59
13	70	700	15	74.93	16.84	23.15	68.78	89.37
14	60	400	15	65.6?	9.79	26.10	55.66	59.08
15	60	700	30	80.87	17.47	35.55	82.43	99.56
16	50	1000	30	62.93	21.55	30.89	76.56	81.64
17	50	700	15	67.92	15.99	28.96	79.63	89.64

All experiments were done in triplicate and the average value of the response variables is given. TPC=total phenol content, TFC=total flavonoid content, TTC=total tannin content, GAE=gallic acid equivalents, QE=quercetin equivalents, TAE=tannic acid equivalents, TE=Trolox equivalnts are expressed on dry mass basis

**Table 3:** Analysis of variance (ANOVA) for assessing the statistical significance of the response variables and the regression coefficients of the second-order model fitted for the *Coccinia grandis* leaf extract

Term	TPC as *w*(GAE)/(mg/g)	TFC as *w*(QE)/(mg/g)	TTC as *w*(TAE)/(mg/g)	DPPH inhibition/%	FRAP as *w*(TE)/(mg/g)
Intercept					
β_0_	82.71	15.63	35.39	82.19	99.47
Linear					
β_1_	3.44*	-0.3216	-1.98**	-3.13**	-1.68*
β_2_	-2.67	5.55**	0.7968	3.64**	-0.4917
β_3_	5.32**	2.17*	2.08**	4.41**	7.33**
Quadratic					
β_11_	-4.84*	2.15	-4.76**	-1.31	1.99
β_22_	-13.93**	-1.47	-5.01**	-16.04**	-25.14**
β_33_	-2.28	0.9146	-2.05*	-2.06	-4.89**
Interaction					
β_12_	-1.65	0.6000	-2.48*	-6.77**	-4.47325**
β_13_	1.26	-1.18	0.5902	1.23	-1.55575
β_23_	0.7780	1.90	-0.1665	0.1735	-2.92325*
CV/%	5.44	11.50	5.02	2.96	2.23
p-value (regression)	0.003**	0.002**	0.0006**	<0.0001**	<0.0001**
p-value (lack of fit)	0.31	0.18	0.38	0.96	0.97

TPC=total phenol content, TFC=total flavonoid content, TTC=total tannin content, GAE=gallic acid equivalents, QE=quercetin equivalents, TAE=tannic acid equivalents, TE=Trolox equivalents are expressed on dry mass basis. *Significant terms, **highly significant terms

A second-order regression model was applied to the data set for TPC and resulted in an R^2^ value of 0.92. This means that approx. 92 % of the variability in the data can be explained by the model. The regression analysis showed that the linear relationship between temperature and extraction time, and the quadratic relationship between temperature and power level had a significant effect on TPC. However, none of the interaction terms proved to be statistically significant (p<0.05). The response surface methodology (RSM) plots illustrating the relationship between the TPC and the independent variables are shown in Fig. 1a. The RSM plots illustrate the relationship between MAE time and temperature and the extraction of TPC. With increasing extraction time and temperature, TPC extraction increased accordingly. On the other hand, when the power was increased, the TPC yield first increased, reached a maximum and then decreased. The experimental results showed that MAE at increased temperatures led to improved extractability of phenolic compounds.

Quantitative analysis of the flavonoid content, expressed as QE, in the leaf extract of *C. grandis* was from 9.79 to 22.99 mg/g. A quadratic regression model was applied to the data set and resulted in an R^2^ value of 0.93. This indicated a higher fit between the model and the observed data, suggesting a more accurate fit of the model. The study found a statistically significant positive correlation (p<0.05) between microwave power and time, suggesting that an increase in these factors led to an increase in the extraction of flavonoids. However, the study did not find any significant effects of other parameters on the extraction of flavonoids. The p-value for the regression analysis was found to be highly significant (p<0.01), indicating strong statistical evidence for the relationship between the variables. Conversely, the p-value for the lack of fit analysis was highly insignificant, suggesting that the model fit the data adequately. The RSM curve, shown in Fig. 1b, shows the influence of microwave power and extraction time on TFC. It demonstrates that with increasing microwave power and extraction time, the yield of flavonoids increased accordingly. The observed increase in yield with longer extraction times can be attributed to an improved mass transport mechanism, which leads to improved extractability (*17*). The result of the experiment showed a negative correlation between temperature and flavonoid extraction efficiency. This indicates the thermosensitivity of *C. grandis* flavonoids. The highest mass fraction of TTC (expressed as TAE on dry mass basis 37.57 mg/g) was observed at a processing temperature of 60 °C, a power of 700 W and the processing time of 30 min. Conversely, the lowest TTC expressed as mass fraction of TAE on dry mass basis (22.65 mg/g) was obtained when the microwave power was set to 1000 W and the processing temperature to 70 °C for 30 min. The regression analysis showed a higher degree of model accuracy, as indicated by an R^2^ value of 0.95. In addition, the p-value of the regression was found to be statistically significant, suggesting a strong relationship between the variables. Additionally, the lack of fit was found to be insignificant, as shown in Table 3. The mathematical expression representing the model fitted to the data set is shown in Table 4. Statistical analysis showed that the linear relationship between extraction time and temperature had a high level of significance (p<0.01). Statistical significance was found for the quadratic coefficients, indicating their influence on the outcome variable. Moreover, the interaction between temperature and power level was also found to be statistically significant, suggesting a combined effect on the outcome variable. The use of RSM curves provides a better visual representation of the effect of independent variables on the TTC. The TTC showed a positive correlation with both temperature and power values. It peaked at intermediate values before decreasing thereafter (Fig. 1c).

**Table 4:** Response variables of biochemical parameters in *Coccinia grandis* leaf extract, based on regression equations and predictor variables

Response	Model	R^2^
TPC	82.71+3.44*x_1_*-2.67*x_2_+*5.32*x_3_*-4.84*x*_1_^2^-13.93*x_2_*^2^-2.28*x_3_*^2^-1.65*x*_12_+1.26*x*_13_-0.77*x*_23_	0.92
TFC	15.63-0.32*x_1_*+5.55*x_2_+*2.17*x_3_*-2.15*x*_1_^2^-1.47*x_2_*^2^+0.91*x_3_*^2^+0.60*x*_12_-1.18*x*_13_+1.90*x*_23_	0.93
TTC	35.39-1.98*x_1_*+0.79*x_2_+*2.08*x_3_*-4.76*x*_1_^2^-5.01*x_2_*^2^-2.05*x_3_*^2^-2.48*x*_12_+0.59*x*_13_+0.16*x*_23_	0.95
DPPH inhibition	82.19-3.13*x_1_*+3.64*x_2_+*4.41*x_3_-*1.31*x*_1_^2^-16.04*x_2_*^2^-2.06*x_3_*^2^-6.77*x*_12_+1.23*x*_13_+0.17*x*_23_	0.98
FRAP	99.47+1.68*x_1_*-0.49*x_2_+*7.3*x_3_*+1.99*x*_1_^2^-25.14*x_2_*^2^-4.89*x_3_*^2^-4.47*x*_12_-1.55*x*_13_-2.92*x*_23_	0.99

TPC=total phenol content, TFC=total flavonoid content, TTC=total tannin content

The evaluation of the antioxidant activity of *C. grandis* extract was carried out under different MAE conditions and the results are shown in Table 2. The DPPH inhibitory activity ranged from 57.22 to 86.39 %, indicating the ability of the tested samples to neutralise free radicals. On the other hand, FRAP values, expressed as TE on dm, ranged from 59.08 to 101.99 mg/g, indicating the ability of the samples to reduce ferric ions. Regression analysis revealed a statistically significant (p<0.01) linear relationship between all independent variables and DPPH activity. The quadratic relationship between microwave power and its effect and the significant interaction between temperature and power were observed. In FRAP, the linear relationship between extraction time and temperature and the quadratic relationship between microwave power and temperature were found to be statistically significant. The correlation between temperature and power showed a statistically significant negative influence (p<0.01), while the influence of power and time was statistically significant (p<0.05). The model showed a high degree of statistical significance, as shown by the p-value of the regression. Additionally, the lack of fit was found to be non-significant, further supporting the suitability of the model for assessing the DPPH and FRAP antioxidant activity. The R^2^ values for the DPPH and FRAP antioxidant activity were determined to be 0.98 and 0.99, respectively. These high R^2^ values indicate that the quadratic models provide a better fit to the data. The RSM plots can be used to improve the understanding of the data, as can be seen in Fig. 1d and Fig. 1e. The observed increase in temperature generally had a detrimental effect on the DPPH and FRAP assays. This phenomenon could be attributed to a decrease in the antioxidant capacity of bioactive compounds with the increase of temperature (*21*). The optimal conditions were determined based on a combined desirability value of 0.862. The optimal solution, which gave the highest values for the desired variables, was achieved at a temperature of 55 °C, a microwave power of 763 W and a processing time of 45 min. This indicates that the extractability of *C. grandis* leaves was improved when a medium temperature and power range with a longer processing time was used. The values of TPC expressed as GAE, TFC as QE, TTC as TAE, and antioxidant activities (DPPH and FRAP as TE) obtained with optimal solution were determined to be 81.87 mg/g, 21.25 mg/g, 35.25 mg/g, 85.84 %, and 102.27 mg/g, respectively. The results were verified by performing experiments covering a range of 10 % deviation from the optimal solution (Table S7). The response variables obtained in the optimal solution showed a high degree of agreement with the optimal solution obtained with predicted RSM values. This is demonstrated by the relative error falling comfortably within the 10 % threshold, proving the effectiveness of the RSM in the optimisation process.

### Screening, purification, identification and characterisation of phytocompounds by chromatography and spectroscopic techniques

**Fig. S2:**
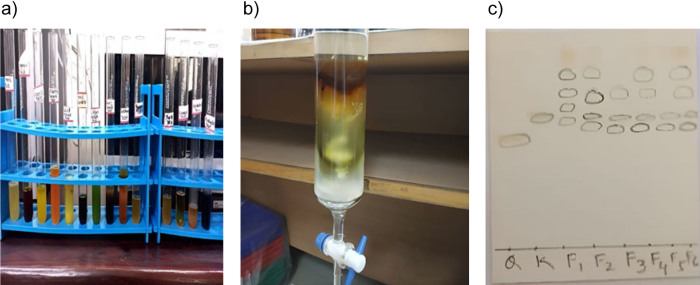
Analysis of phytochemicals from *Coccinia grandis* leaf extract included three main steps: a) preliminary phytochemical screening, b) column chromatography, and c) identification of the presence of kaempferol by thin-layer chromatography

The phytocompounds were obtained by isolation from different fractions of extracts, which were then compared with the standard compounds quercetin and kaempferol. The spots were then detected and identified in the UV-Vis spectrum. A total of six different spots were detected on the TLC plate. It was expected that one of these spots would correspond to kaempferol, as shown in Fig. S2. The results of this study confirm the findings of Mohanty *et al.* (*46*).

**Fig. S3:**
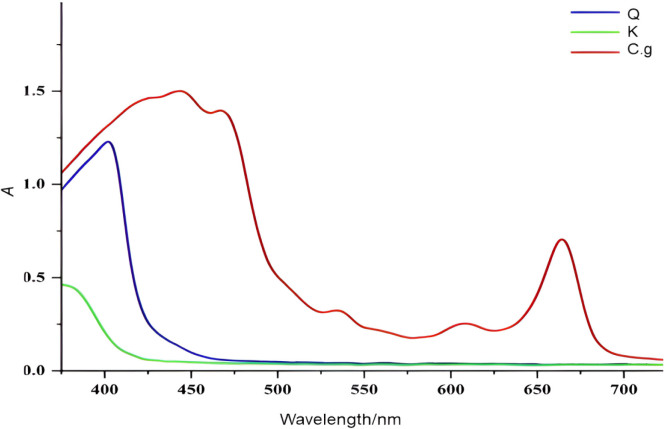
Spectra for the presence of phenolic compounds and flavonoids in *Coccinia grandis* leaf extract using UV-visible spectral analysis. K=kaempferol, Q=quercetin, C.g=*Coccinia grandis*

The presence of an aromatic ring, together with other rings, in phenolic and flavonoid compounds was responsible for the manifestation of two different absorption spectra. The absorption peak observed at a wavelength of 285 nm represents the π-π* transitions that occur within the aromatic system. The absorption peak observed between 300 and 600 nm corresponds to the transitions that occur within a different ring structure. It is believed that the appearance of a secondary peak in the sample is a result of the superposition of ligand-to-metal charge transfer (LMCT) bands. Due to this feature, the UV-Vis spectroscopy effectively identified the chromophore moieties in the isolated molecules known from various botanical extracts. The plant extract was subjected to qualitative UV-Vis analysis to identify and characterise compounds containing σ-bonds, π-π* bonds, lone pairs of electrons, aromatic rings and chromophore groups. The obtained results were compared with the UV-Vis profiles of quercetin and kaempferol. Both standards show two absorption peaks in the 400 nm wavelength range, indicating the presence of organic chromophores and suggesting the existence of phenols and flavonoids in the leaf extract (Fig. S3). These observations are in agreement with the conclusions of an earlier study by Nowak *et al.* (*47*).

**Fig. 2:**
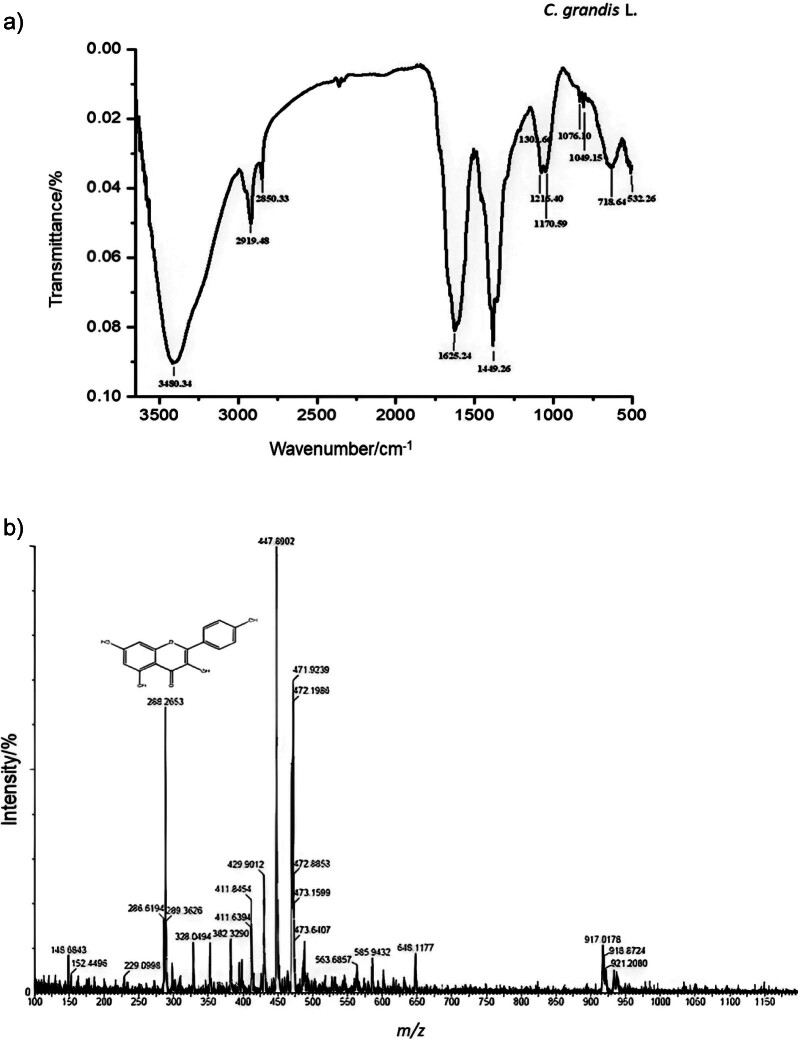
Two different spectra of the leaf extract of *Coccinia grandis* obtained using: a) Fourier transform infrared (FTIR) spectroscopy and b) liquid chromatography-mass spectrometry (LC-MS) specifically show the presence of kaempferol

The observed FTIR spectra of *C. grandis* leaves show the absorption frequencies and intensities associated with each band. The extract contains a significant amount of flavonoids and other phenolic compounds, most of which contain a hydroxyl group. This feature explains the observed broad absorption frequency of 3480 cm^-1^. The presence of methoxy, methylene and methyl functional groups resulted in the stretching of −CH bonds at a frequency of 2919 cm^-1^. However, a slightly lower frequency of 2850 cm^-1^ was observed in the phytocompounds due to the presence of alkanes. The presence of −C=C functional groups in all compounds led to the observed peak at 1625 cm^-1^. On the other hand, the peaks at 1449 and 1301 cm^-1^ could possibly be due to deformations involving −NH_3_ and −CH_3_ as well as other compounds containing −NH and −CH_3_ groups, respectively. The analysis of phytocompounds showed that the stretching of the C−O bond occurred at different peak positions: 1216, 1170, 1076 and 1049 cm^-1^. Additionally, the bending of the C−C bond leads to vibrational peaks at 718 and 532 cm^-1^, as shown in Fig. 2. These results are in good agreement with the results of Sharma *et al.* (*28*).

The results of LC-MS analysis show different peaks at different retention times, indicating the presence of different phytochemicals. These peaks were further characterised by their corresponding mass-to-charge ratio (*m/z*) values and prominent compounds were successfully identified based on their retention time. Compounds such as benzafuranone (*m/z*=136.36), ethisterone (*m/z*=310.14), ferulic acid (*m/z*=194.63), luteolin (*m/z*=286.20), camptothecin (*m/z*=348.70) and conifyryl alcohol (*m/z*=180.59) were detected and characterised at specific retention times of 5.6, 11.60, 12.63, 28.04 and 30.34 min, respectively. Kaempferol (*m/z*=286.61) (Fig. 2b) and luteolin (*m/z*=286.20), two prominent flavonoid compounds, were successfully characterised based on their retention times of 15.10 and 17.22 min, respectively. Table S8 contains a comprehensive list of compounds detected by LC-MS, together with their respective retention times and *m/z* values.

Preliminary screening, column chromatography and TLC analyses indicated the presence of alkaloids and phenols, which were identified as the major constituents. The results were additionally corroborated by UV-visible and FTIR spectroscopic analyses. These results are in agreement with previous studies by Prabhakar *et al.* (*39*, *48*). Subsequently, a total of 16 chemical compounds were detected and characterised by LC-MS. Among these compounds, phenolic compounds such as kaempferol and luteolin were found. The compounds detected by LC-MS analysis in the present study were in agreement with the findings of Al-Madhagy *et al.* (*31*).

### Evaluation of the inhibitory capacity of C. grandis leaf extract by in vitro assessment of the inhibition of α-amylase and α-glucosidase enzymes

A significant inhibitory effect was observed when the *in vitro* α-amylase inhibitory capacity of the *C. grandis* leaf extract was tested. When acarbose and leaf extract were compared, it was observed that the inhibition of α-amylase by the leaf extract increased linearly but gradually when the concentration increased up to 31.2 µg/mL. Consequently, the concentration of the leaf extract showed an increasing trend in correlation with the suppression of α-amylase enzymes. The IC_50_ value of (52.4±2.7) µg/mL, which was higher than the inhibitory concentration of the standard acarbose, was determined as the maximum inhibitory concentration ((70.8±1.2) %) at a concentration of 125.0 µg/mL (Table S9). In a study conducted by Pulbutr *et al.* (*7*), the aqueous leaf extract of *C. grandis* was observed to have a significant inhibitory effect on the α-amylase enzyme, as evidenced by an IC_50_ value of (8.1±0.7) µg/mL. In another study, Putra *et al.* (*49*) observed that the plant sample showed the strongest inhibition of α-amylase activity, with the lowest half maximal inhibitory concentration (IC_50_=0.8±0.4 mg/mL). Packirisamy and Sivaprakasam (*50*) reported that the fruit extract of *C. grandis* showed significant inhibitory effect on α-amylase. The maximum inhibition, reaching (62.2±3.4) %, was observed at a concentration of 250 µg/mL and IC_50_ value of (117.6±4.5) µg/mL.

**Fig. S4:**
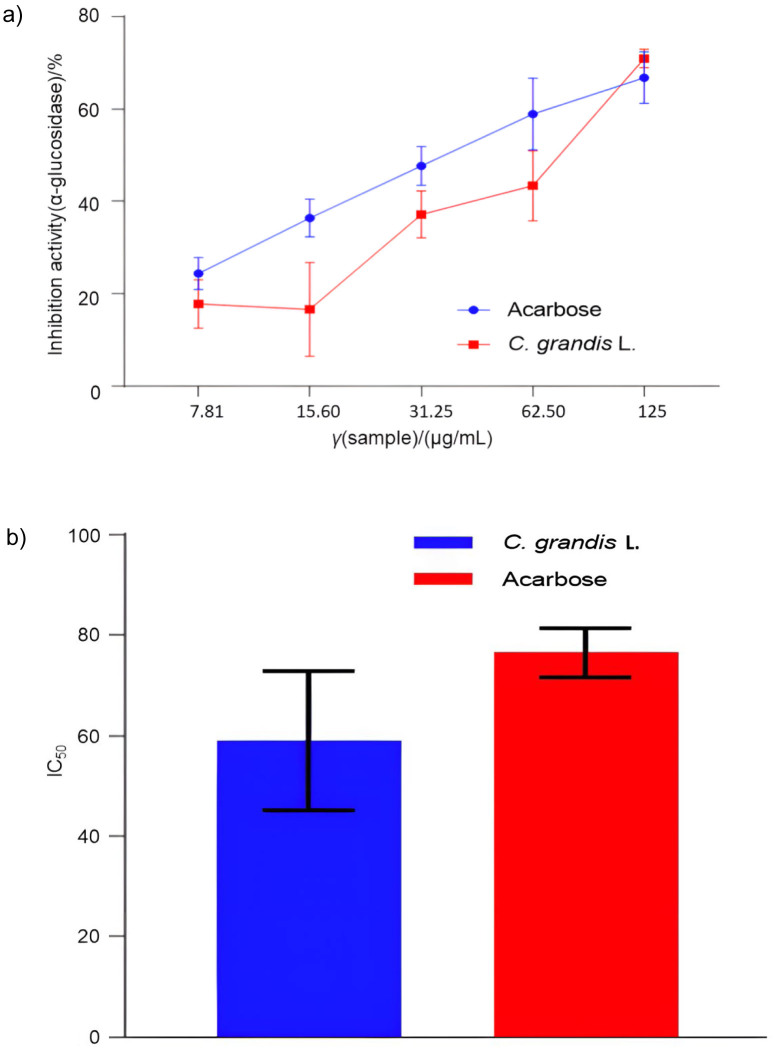
Determination of inhibitory activity of *Coccinia grandis* leaf extract and acarbose on α-glucosidase: a) inhibition percentage (maximum value of (70.9±1.6) %) at different concentrations of the leaf extract, and b) its half maximal inhibitory concentration (IC_50_=(76.5±4.0) μg/mL). All results are given as mean values of three experiments (*N*=3, p<0.05)

The ethanolic extract of *C. grandis* showed no inhibitory effect up to a concentration of 15.62 µg/mL compared to acarbose. However, as the concentration increased at each data point, significant growth was observable. The maximum inhibition of (70.9±1.6) % was observed at a concentration of 125.0 µg/mL, which exceeded the percentage of inhibition of the reference antidiabetic drug acarbose (Fig. S4a). The IC_50_ value of the ethanolic extract of *C. grandis* leaves was determined to be (76.5±4.0) µg/mL, showing a lower inhibitory effect compared to acarbose (Fig. S4b). In addition, the IC_50_ values were determined at a lower concentration and exceeded the values observed in previous studies (*7*, *48*). In another study by Packirisamy and Sivaprakasam (*50*), the extract obtained from *C. grandis* was shown to have an inhibitory effect on α-glucosidase at a concentration of 250 µg/mL. The maximum inhibition achieved was (67.6±2.8) %, while acarbose showed a higher inhibition rate of (87.3±4.6) %. The IC_50_ value of the *C. grandis* extract was (81.6±3.6) µg/mL, while α-glucosidase inhibitors such as acarbose had a lower IC_50_ value of (44.5±2.3) µg/mL. Based on the existing literature and recent research, it can be concluded that the extract obtained from *C. grandis* has α-glucosidase enzymatic activity. The presence of phytoconstituents in the extracts could be a reason for the observed ability to inhibit enzymes *in vitro*.

### Evaluation of the cytotoxicity of C. grandis leaf extract by in vitro and ex vivo toxicity tests

**Fig. 3:**
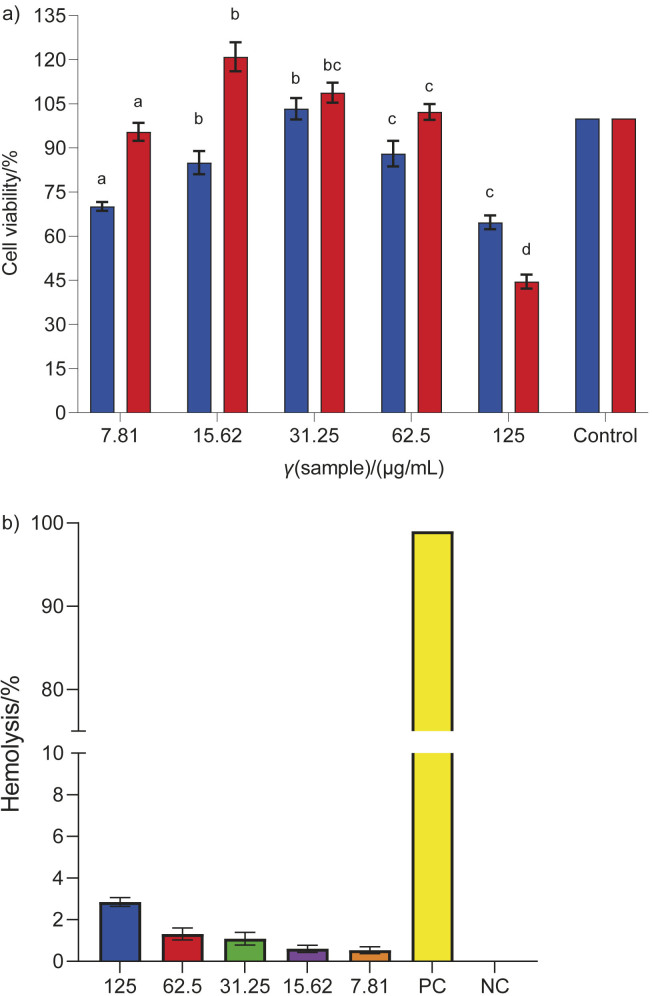
Effect of different concentrations of *Coccinia grandis* leaf extract on: a) cell viability at different concentrations of the leaf extract compared to untreated control after 24 (blue) and 72 h (red) and b) haemolytic activity (~3.08 % haemolysis) of the leaf extract. PC=positive control, NC=negative control

Different concentrations of *C. grandis* ethanolic extract, ranging from 7.8 to 125.0 µg/mL, were administered to L929 fibroblast cells for 24 and 72 h. After 24 h of incubation, cell viability was significantly reduced at concentrations of 7.8 and 15.6 µg/mL. However, at a concentration of 31.2 µg/mL, the cell viability was considerably higher. When the concentration reached 62.5 µg/mL, it decreased, and at a concentration of 125.0 µg/mL, it was exceptionally low. The plant extract also showed the lowest cell viability at a concentration of 125.0 µg/mL after 72 h, suggesting that cytotoxic stress was present in the specimens at concentrations above 62.5 µg/mL (Fig. 3a). In the present study, the leaf extract of *C. grandis* showed the lowest cytotoxicity to the cell lines at concentrations up to 62.5 µg/mL. The results suggest that the samples are cytocompatible within a specific concentration range, but the compounds extracted from them may be cytotoxic at higher concentrations (*39*, *48*). In a study by Bunkrongcheap *et al.* (*51*), it was reported that the application of stem and leaf extracts did not have any discernible effect on the differentiation of 3T3-L1 adipocytes. The observed reduction in lipid levels at a concentration of 400 μg/mL of leaf extract and 800 μg/mL of stem extract can be attributed to their cytotoxic effect, as shown by the results of the MTT assay. Based on the results of the MTT experiment, it was observed that pretreatment with *C. grandis* extract led to a significant decrease in the detrimental effects of alloxan-induced RINm5F cell lysis (*52*). Compared to cells treated solely with alloxan, the application of the extract resulted in a significant increase in cell viability, which reached (79.4±3.7) % at a concentration of 0.50 mg/mL. To mitigate possible negative effects on cell viability, the concentration of the extract was limited to 0.50 mg/mL, as described by Meenatchi *et al.* (*52*). In the study by Varma and Bhaskar (*53*), the researchers determined the effective concentration of *C. grandis* extract by analysing the dose-response curve and calculating the IC_50_ values. For this purpose, the researchers used different concentrations of the extract (2, 5, 10, 20, 40, 80, 100 and 200 µg/mL). The ethyl acetate fraction obtained from *C. grandis* did not show significant cytotoxic effects, as determined by the percentage of cytotoxicity compared to the reference treated with the drug.

**Fig. 4:**
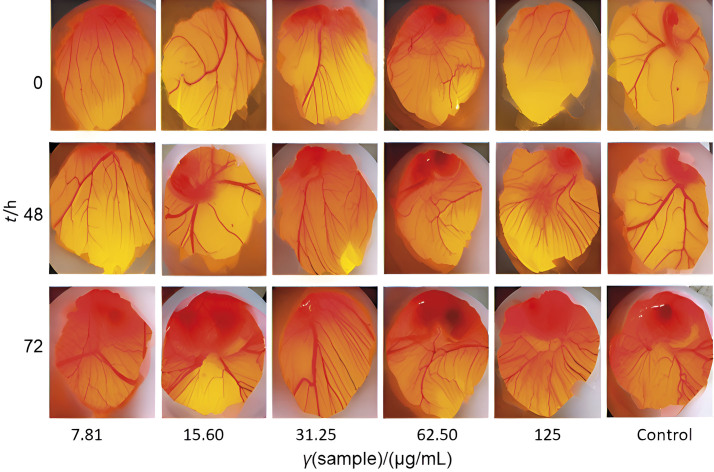
The chorioallantoic membrane (CAM) test was performed with the leaf extracts of *Coccinia grandis*. An ethanol leaf extract was administered to the eggs at concentrations from 7.8 to 125.0 μg/mL over a period of 0, 48 and 72 h. There was no evidence of suppression of vascularisation in the sample at none of the concentrations up to 125.0 μg/mL. The attenuation and degeneration of blood vessels, which were not observed at any concentration, are indicative of toxic effects

The induction of interference with red blood cells is essential for understanding the host immune response following exposure to foreign substances in order to elicit immunogenic response. As shown in Fig. 3b, the haemocompatibility of *C. grandis* (at concentrations ranging from 7.8 to 125.0 μg/mL) was detected with a maximum haemolysis rate of ~3.08 %. The haemolysis assay is a valuable tool to evaluate the potential link between cytotoxic activity and direct disruption of the cell membrane. The plant extract showed a significantly low haemolytic activity of 3.8 % (*54*, *55*). This finding is a prof of strong compatibility with cell membranes and red blood cells. Zohra and Fawzia (*54*) performed haemolytic tests on a range of plant species and found that most plants did not have haemolytic activity. In another study, Majumder *et al.* (*56*) found that the concentration tests of *C. grandis* extract did not show any haemolytic activity against human erythrocytes. When the concentration of crude extracts of *C. grandis* was increased from 250 to 500 µg/mL, no observable changes in haemolytic activity on the membrane of blood erythrocytes were observed. At concentrations of 250 and 500 µg/mL, the observed haemolytic activities were 0.23 and 0.45 %, respectively. The CAM experiment showed that the use of the plant extract at concentrations from 7.8 to 125.0 µg/mL did not exhibit any adverse effects on angiogenesis or embryonic growth in the avian model. At intervals of 0, 48 and 72 h, as shown in Fig. 4, the blood capillaries were observed to continuously expand with simultaneous thickening of the membrane walls. With increasing concentration of *C. grandis* leaf extract, there was no observable inhibition of angiogenesis in the CAM of the chicken embryo. In animal models conducted *in vitro*, it was observed that the aqueous extracts from leaves did not induce any pathological morphological changes in the tissues. Moreover, these extracts did not show any detrimental effects on the function of vital organs such as liver, kidney or bone marrow, as shown by Sankarganesh *et al.* (*57*).

## CONCLUSIONS

The α-amylase and α-glucosidase inhibitors have been shown to effectively mitigate postprandial hyperglycaemia, making them valuable therapeutic drugs for the treatment of diabetes. The consumption of plant foods rich in polyphenolic and flavonoid compounds has the potential to prevent up to 90 % of diabetes cases. In particular, the leaves of *Coccinia grandis* have been identified as a valuable source of phenolic compounds. The identification of α-amylase and α-glucosidase inhibitors of plant origin therefore represents a highly effective strategy to mitigate adverse reactions and reduce the financial burden. In the present study, we evaluated the antidiabetic and cytocompatible phytochemicals found in the leaf extracts of *C. grandis* using *in silico*, *in vitro* and *ex vivo* approaches. The optimum conditions of 55 °C, 763 W and 45 min had a significant effect on the total phenolic, total flavonoid and total tannin contents, percentage of DPPH inhibition and FRAP assays and resulted in a high recovery. The study showed that the phytocompounds obtained from *C. grandis* had a remarkable inhibitory effect on the activity of α-amylase and α-glucosidase. This inhibition was observed to depend on the concentration of the phytocompounds. The results of the MTT assay showed that the extracts were cytocompatible up to a concentration of 62.5 µg/mL. The low haemolytic activity observed further indicates the high cellular biocompatibility of the substance. In addition, the CAM assay showed no signs of cytotoxicity in all measured concentration ranges of the plant extract. Therefore, the current study suggests the use of *C. grandis* leaves in the development of antidiabetic drugs and functional food products as an effective therapeutic and nutraceutical approach for the treatment of diabetes mellitus.

## Appendix

**Table S1:** Protein Data Bank (PDB) identifiers, central grid values and resolutions of enzymes used in the study

Enzyme	PDB ID	Centre grid (X, Y, Z)/Å	Resolution (X-ray diffraction)/Å	Exhaustiveness
α-amylase	3bc9	47.788286, 25.075619, 5.722952	1.35	100
α-glucosidase	1obb	30.179130, 33.953391, 22.922000	1.90	100

**Table S2:** Absorption and distribution properties of phytochemicals from the leaf extract of *Coccinia grandis* to assess their likely permeability and bioavailability across different physiological barriers

Phytochemical	HIA	Caco-2 permeability	BBBP	P-glycoprotein inhibitor	P-glycoprotein substrate	MDCK permeability	F_20 %_	F_30 %_	PPB/%	VD	Fu/%
Benzofuranone	0.006	−4.594	0.239	0.001	0.255	2.2e-05	0.888	0.906	92.28	0.849	10.170
Campesterol	0.004	−4.740	0.854	0.377	0.001	9e-06	0.013	0.243	98.61	1.842	1.789
Campocatechin	0.004	−4.930	0.529	0.013	0.005	2.7e-05	0.002	0.003	90.80	0.525	3.310
Coniferyl alcohol	0.010	−4.457	0.295	0.001	0.321	1.8e-05	0.005	0.742	80.73	1.059	16.950
Ethisterone	0.008	−4.725	0.964	0.104	0.000	2.1e-05	0.553	0.004	95.28	1.034	1.424
Ferulic acid	0.030	−4.902	0.329	0.000	0.086	1.5e-05	0.047	0.584	89.75	0.339	6.394
Furanone	0.006	−4.446	0.219	0.000	0.022	2.2e-05	0.015	0.008	79.21	2.455	38.780
Isosteviol	0.006	−5.285	0.221	0.009	0.000	1.7e-05	0.002	0.890	91.25	0.558	9.029
Kaempferol	0.008	−4.974	0.009	0.004	0.001	9e-06	0.856	0.993	97.86	0.522	4.411
Luteolin	0.047	−5.028	0.009	0.004	0.274	1e-05	0.998	1.000	95.43	0.533	5.984
Methyl caffeate	0.020	−4.631	0.185	0.001	0.077	2e-05	0.025	0.806	85.83	0.390	10.410
*p*-coumaric acid	0.009	−4.961	0.290	0.000	0.017	1.3e-05	0.004	0.194	85.36	0.293	13.720
Quercetin	0.014	−5.204	0.008	0.004	0.005	8e-06	0.930	0.997	95.49	0.579	7.423
Listroside	0.927	−5.702	0.596	0.000	0.673	0.000102	0.100	0.998	63.72	0.394	34.460
Lukianol	0.007	−4.744	0.010	0.048	0.006	1.3e-05	0.943	0.537	101.10	0.414	0.795
Oleuropein	0.950	−5.847	0.672	0.000	0.842	0.000103	0.371	0.998	74.00	0.568	25.640
Sinapic acid	0.165	−4.962	0.271	0.001	0.040	1.3e-05	0.189	0.624	88.79	0.450	9.389
Stigmasterol	0.005	−4.668	0.691	0.066	0.001	8e-06	0.008	0.185	98.67	2.408	1.572
Undecanol	0.003	−4.414	0.890	0.004	0.012	2.2e-05	0.261	0.979	93.51	1.628	5.165

HIA=human intestinal absorption: category 1=HIA+(HIA <30 %), category 0=HIA-(HIA <30 %). Caco-2 permeability: optimal=higher than -5.15 log cm/s. BBBP=blood-brain barrier penetration: category 1=BBB+, category 0=BBB-. P-glycoprotein inhibitor: category 1=inhibitor, category 0=non-inhibitor. P-glycoprotein substrate: category 1=substrate, category 0=non-substrate. MDCK permeability=Madin−Darby canine kidney cells: low permeability: <2·10^-6^ cm/s, medium permeability: 2 to 20·10^-6^ cm/s, high passive permeability: >20·10^-6^ cm/s. F_20 %_=20 % bioavailability: category 1=F_20 %_+ (bioavailability <20 %), category 0=F_20 %_- (bioavailability ≥20 %). F_30 %_=30 % bioavailability: category 1=F_30 %_+ (bioavailability <30 %), category 0=F_30 %_- (bioavailability ≥30 %). PPB=plasma protein binding: optimal <90 %, Highly protein-bound drugs may have a low therapeutic index. VD=volume distribution: optimal=0.04-20 L/kg. Fu=fraction unbound: in plasma, low <5 %, medium 5-20 %, high >20 %. The output value is the probability of being within the range of 0 to 1

**Table S3:** The metabolic profile of phytochemicals from *Coccinia grandis* leaf extract with different substrates and inhibitors of the cytochrome P450 enzyme family

Phytochemical	CYP1A2 inhibitor	CYP1A2 substrate	CYP2C19 inhibitor	CYP2C19 substrate	CYP2C9 inhibitor	CYP2C9 substrate	CYP2D6 inhibitor	CYP2D6 substrate	CYP3A4 inhibitor	CYP3A4 substrate
Benzofuranone	0.914	1.716	0.156	0.091	0.044	0.776	0.161	0.774	0.018	0.215
Campesterol	0.055	0.523	0.071	0.957	0.093	0.216	0.005	0.445	0.181	0.769
Campocatechin	0.928	0.767	0.629	0.346	0.554	0.429	0.054	0.458	0.298	0.419
Coniferyl alcohol	0.709	0.867	0.042	0.422	0.032	0.836	0.019	0.893	0.087	0.241
Ethisterone	0.107	0.846	0.891	0.93	0.445	0.23	0.093	0.142	0.639	0.93
Ferulic acid	0.059	0.478	0.042	0.057	0.142	0.367	0.02	0.199	0.026	0.075
Furanone	0.205	0.147	0.048	0.072	0.014	0.399	0.018	0.597	0.008	0.179
Isosteviol	0.005	0.855	0.016	0.901	0.089	0.254	0.007	0.157	0.26	0.078
Kaempferol	0.972	0.11	0.181	0.046	0.653	0.867	0.722	0.283	0.697	0.08
Luteolin	0.981	0.154	0.124	0.046	0.576	0.842	0.568	0.559	0.549	0.092
Methyl caffeate	0.899	0.696	0.252	0.078	0.436	0.86	0.164	0.732	0.292	0.227
*p*-coumaric acid	0.061	0.067	0.046	0.052	0.232	0.566	0.019	0.169	0.031	0.08
Quercetin	0.943	0.115	0.053	0.041	0.598	0.643	0.411	0.205	0.348	0.046
Listroside	0.014	0.077	0.045	0.204	0.016	0.145	0.012	0.084	0.21	0.2
Lukianol	0.885	0.104	0.769	0.043	0.491	0.889	0.289	0.871	0.308	0.23
Oleuropein	0.017	0.071	0.035	0.071	0.014	0.582	0.003	0.12	0.357	0.157
Sinapic acid	0.046	0.833	0.026	0.063	0.053	0.277	0.016	0.198	0.01	0.084
stigmasterol	0.041	0.603	0.074	0.954	0.106	0.132	0.038	0.643	0.339	0.852
Undecanol	0.905	0.319	0.401	0.07	0.281	0.865	0.01	0.077	0.058	0.074

Obtained values provide information on the specific metabolic pathways and interactions involved in the biotransformation of the phytochemicals. The output value is the probability of being substrate/inhibitor, within the range of 0 (no effect) to 1 (high effect)

**Table S4:** Toxicological effects of phytochemicals from *Coccinia grandis* on the human body based on different toxicity tests

Phytochemical	hERG blocker	Hepatotoxicity	DILI	Ames toxicity	Rat oral acute toxicity	FDAMDD	Skin sensitisation	Carcinogenicity	Eye corrosion	Eye irritation	Respiratory toxicity
Benzofuranone	0.013	0.582	0.582	0.266	0.422	0.032	0.391	0.835	0.742	0.991	0.638
Campesterol	0.04	0.193	0281	0.032	0.023	0.645	0.176	0.067	0.003	0.01	0.502
Campocatechin	0.162	0.753	0.414	0.744	0.876	0.908	0.04	0.562	0.003	0.014	0.036
Coniferyl alcohol	0.035	0.17	0.045	0.141	0.386	0.09	0.949	0.662	0.493	0.987	0.502
Ethisterone	0.041	0.149	0.18	0.015	0.163	0.884	0.032	0.916	0.004	0.023	0.961
Ferulic acid	0.023	0.345	0.511	0.114	0.733	0.076	0.929	0.443	0.515	0.979	0.72
Furanone	0.02	0.13	0.126	0.071	0.877	0.016	0.293	0.908	0.985	0.996	0.906
Isosteviol	0.002	0.422	0.027	0.033	0.082	0.132	0.012	0.102	0.004	0.507	0.949
Kaempferol	0.07	0.098	0.979	0.672	0.156	0.109	0.856	0.097	0.009	0.929	0.09
Luteolin	0.064	0.084	0.905	0.536	0.046	0.741	0.946	0.095	0.009	0.944	0.22
Methyl caffeate	0.034	0.244	0.082	0.375	0.801	0.234	0.94	0.392	0.046	0.461	0.448
*p*-coumaric acid	0.025	0.673	0.2	0.045	0.796	0.031	0.941	0.151	0.672	0.988	0.512
Quercetin	0.099	0.1	0.98	0.657	0.065	0.31	0.919	0.05	0.007	0.936	0.072
Listroside	0.005	0.168	0.954	0.693	0.088	0.147	0.032	0.954	0.003	0.007	0.643
Lukianol	0.106	0.25	0.976	0.012	0.1	0.385	0.206	0.099	0.003	0.106	0.064
Oleuropein	0.003	0.127	0.954	0.781	0.136	0.2	0.028	0.953	0.003	0.007	0.72
Sinapic acid	0.018	0.363	0.21	0.016	0.321	0.055	0.949	0.278	0.391	0.94	0.428
Stigmasterol	0.012	0.011	0.055	0.029	0.054	0.539	0.025	0.054	0.003	0.001	0.19
Undecanol	0.106	0.016	0.04	0.007	0.041	0.013	0.926	0.066	0.991	0.971	0.341

hERG=human ether-a-go-go related gene, DILI=drug-induced liver injury, FDAMDD=Food and Drug Administration recommended maximum daily drug dose. All values are between 0 and 1, with 0 indicating that there is no effect and 1 that there is a high effect

**Table S5:** Mechanism for predicting the toxicity of *Coccinia grandis* leaf extract using the Tox21 pathway to evaluate the effects of phytochemicals on both nuclear and stress response pathways

Phytochemical	NR-AR	NR-AR-LBD	NR-AhR	NR-aromatase	NR-ER	NR-ER-LBD	NR-PPAR-γ	SR-ARE	SR-ATAD5	SR-HSE	SR-MMP	SR-p53
Benzofuranone	0.013	0.005	0.375	0.01	0.29	0.008	0.004	0.528	0.016	0.06	0.346	0.067
Campesterol	0.002	0.003	0.0	0.012	0.397	0.933	0.008	0.123	0.001	0.046	0.89	0.014
Campocatechin	0.027	0.626	0.625	0.905	0.489	0.256	0.808	0.86	0.6	0.715	0.851	0.97
Coniferyl alcohol	0.033	0.014	0.613	0.08	0.152	0.011	0.011	0.482	0.176	0.239	0.317	0.422
Ethisterone	0.765	0.919	0.005	0.589	0.671	0.865	0.563	0.699	0.045	0.588	0.965	0.855
Ferulic acid	0.809	0.278	0.277	0.021	0.254	0.033	0.07	0.525	0.19	0.296	0.22	0.171
Furanone	0.029	0.003	0.017	0.004	0.168	0.006	0.002	0.073	0.006	0.019	0.023	0.005
Isosteviol	0.265	0.044	0.003	0.578	0.132	0.061	0.858	0.135	0.045	0.239	0.571	0.091
Kaempferol	0.008	0.371	0.967	0.941	0.959	0.985	0.963	0.873	0.612	0.557	0.968	0.921
Luteolin	0.079	0.169	0.977	0.908	0.954	0.996	0.939	0.884	0.675	0.888	0.976	0.891
Methyl caffeate	0.735	0.655	0.912	0.195	0.572	0.855	0.375	0.905	0.8	0.892	0.827	0.796
*p*-coumaric acid	0.857	0.349	0.229	0.011	0.477	0.432	0.012	0.783	0.153	0.237	0.318	0.347
Quercetin	0.01	0.179	0.967	0.917	0.927	0.987	0.961	0.815	0.436	0.655	0.962	0.888
Listroside	0.006	0.241	0.035	0.032	0.186	0.013	0.048	0.161	0.202	0.017	0.0715	0.44
Lukianol	0.078	0.567	0.915	0.965	0.981	0.996	0.975	0.948	0.706	0.482	0.989	0.93
Oleuropein	0.006	0.185	0.111	0.044	0.187	0.011	0.251	0.329	0.298	0.06	0.728	0.42
Sinapic acid	0.226	0.153	0.205	0.393	0.198	0.013	0.829	0.57	0.483	0.357	0.28	0.678
stigmasterol	0.0	0.002	0.0	0.005	0.369	0.923	0.005	0.135	0.001	0.052	0.943	0.015
Undecanol	0.016	0.002	0.006	0.041	0.0288	0.017	0.04	0.066	0.004	0.519	0.051	0.031

NR-AR=nuclear-hormone receptor, androgen receptor, NR-AR-LBD=nuclear-hormone receptor, androgen receptor-ligand binding domain, NR-AhR=nuclear-hormone receptor-aryl hydrocarbon receptor, NR-ER=nuclear hormone receptor-estrogen receptor, NR-ER-LBD=nuclear hormone receptor-estrogen receptor ligand-binding domain, NR-PPAR-γ=nuclear hormone-peroxisome proliferator-activated receptor gamma, SR-ARE=antioxidant response element, SR-ATAD5=ATPase family AAA domain-containing protein 5, SR-HSE=heat shock factor response element, SR-MMP=mitochondrial membrane potential, SR-p53. All the values are between 0 and 1, with 0 indicating that there is no effect and 1 that there is a high effect

**Table S6:** Results of the process of elimination and investigation of the harmful effects of phytochemicals from *Coccinia grandis* on the external environment to understand their excretion dynamics and environmental toxicology

					LC_50_	
Phytochemical	Clearance/(mL/(min·kg)	*t*_1/2_	BCF/log10 (L/kg)	IC_50_	PP	DM
Benzofuranone	11.101	0.83	0.718	2.999	3.449	4.352
Campesterol	17.948	0.0015	3.141	4.855	4.972	5.897
Campocatechin	6.862	0.332	1.379	4.15	6.075	5.553
Coniferyl alcohol	12.341	0.92	0.707	2.958	3.625	3.913
Ethisterone	5.594	0.208	0.559	2.81	2.71	4.556
Ferulic acid	7.48	0.926	0.454	3.086	3.249	3.908
Furanone	14.22	0.894	0.522	2.235	2.799	3.76
Isosteviol	0.344	0.503	0.212	3.28	3.215	4.207
Kaempferol	6.868	0.905	0.986	4.386	5.223	5.205
Luteolin	8.146	0.898	1.016	4.432	5.222	5.302
Methyl caffeate	15.624	0.93	0.657	3.886	0.047	4.92
*p*-coumaric acid	6.299	0.919	0.416	3.136	3.373	3.775
Quercetin	8.284	0.929	1.017	4.231	5.222	5.331
Listroside	2.057	0.647	0.519	3.099	4.572	5.312
Lukianol	6.776	0.276	1.856	5.61	6.248	6.491
Oleuropein	2.585	0.832	0.556	3.52	4.591	5.383
Sinapic acid	7.776	0.933	0.472	2.765	3.002	3.951
stigmasterol	15.958	0.014	3.367	4.978	5.511	6.355
Undecanol	7.769	0.409	1.871	4.853	4.811	3.82
Clearance: high >15, moderate 5–15, low <5 mL/(min·kg); *t*_1/2_=half-life: category 1=long half-life (>3 h), category 0=short half-life (<3 h). BCF=bioconcentration factors used for considering secondary poisoning potential and assessing risks to human health *via* the food chain. IC_50_=50 % growth inhibitory concentration on *Tetrahymena pyriformis*, LC_50_=50 % lethal concentration on *Pimephales promelas* (PP) and *Daphnia magna* (DM) after 96 and 48 h, respectively						

**Table S7:** Predicted and validated values of total phenolic content (TPC), total flavonoid content (TFC), total tannin content (TTC), 2,2-diphenyl-1-picrylhydrazyl (DPPH) radical scavenging activity, and Fe(III) reducing antioxidant power (FRAP) expressed on dry mass basis obtained with the optimized conditions of microwave-assisted extraction of *Coccinia grandis* leaf extract

Parameter	Experimental value	RSM value	Relative error/%
TPC as *w*(GAE)/(mg/g)	79.86	81.87	2.4
TFC as *w*(QE)/(mg/g)	23.12	21.24	8.85
TTC as *w*(TAE)/(mg/g)	35.03	35.24	0.59
DPPH/%	83.62	85.83	2.5
FRAP as *w*(TE)/(mg/g)	101.56	102.27	0.69

RSM=response surface methodology, GAE=gallic acid equivalents, QE=quercetin equivalents, TAE=tannic acid equivalents, TE=Trolox equivalents

**Table S8:** Identification of phytochemicals from *Coccinia grandis* leaf using liquid-chromatography-mass spectrometry analysis, their mass-to-charge ratios (*m*/*z*) and corresponding retention times (*t*_R_)

Sample	Phytocompound	Molecular formula	Compound type	*t*_R_/min	Parent ion (*m/z*)	Ionization mode
1	Benzofuranone	C_8_H_6_O_2_	Furan	5.6	136.36	Negative
2	Camptothecin	C_20_H_16_N_2_O_4_	Quinoline	28.04	348.70	Negative
4	Coniferyl alcohol	C_10_H_12_O_3_	Alcohol	30.34	180.54	Negative
5	Ethisterone	C_10_H_12_O_3_	Steroid	11.60	310.14	Negative
6	Ferulic acid	C_10_H_10_O_4_	Cinnamic acid	12.63	194.63	Negative
7	Isosteviol	C_20_H_30_O_3_	Terpenoid	39.58	316	Negative
8	Kaempferol	C_15_H_10_O_6_	Flavonoid	15.10	286.16	Positive
9	Luteolin	C_15_H_10_O_6_	Flavone	17.22	286.20	Positive
10	Methyl caffeate	C_10_H_10_O_4_	Caffeate ester	14.67	194.70	Negative
11	*p*-coumaric acid	C_9_H_8_O_3_	Coumarin	3.95	164.15	Positive
12	Sinapic acid	C_11_H_12_O_5_	Cinnamic acid	20.34	224.71	Negative
13	Stigmasterol	C_29_H_48_O	Sterol	27.01	412.05	Negative
14	Undecanol	C_11_H_24_O	Alcohol	1.09	172.64	Negative
15	Isosteviol	C_20_H_30_O_3_	Terpenoid	39.58	316	Negative
16	Kaempferol	C_15_H_10_O_6_	Flavonoid	15.10	286.16	Positive

**Table S9:** Inhibitory effect of *Coccinia grandis* extract at different concentrations on α-amylase, and its IC_50_ value

	α-amylase inhibition/%	
*γ*(extract)/(µg/mL)	Acarbose	*C. grandis*
125.0	56.4±2.4	70.8±1.2
62.5	53.3±3.4	52.6±0.7
31.2	42.7±3.2	46.3±2.0
15.6	33.7±1.8	43.1±1.1
7.8	26.7±2.5	30.3±0.8
IC_50_/(µg/mL)	79.9±8.1	52.4±2.7

The results are expressed as mean value±standard deviation, *N*=3, p<0.05
